# Generalized joint hypermobility in childhood is a possible risk for the development of joint pain in adolescence: a cohort study

**DOI:** 10.1186/s12887-014-0302-7

**Published:** 2014-12-10

**Authors:** Oline Sohrbeck-Nøhr, Jens Halkjær Kristensen, Eleanor Boyle, Lars Remvig, Birgit Juul-Kristensen

**Affiliations:** Institute of Sports Science and Clinical Biomechanics, University of Southern Denmark, Campusvej 55, DK-5230 Odense, Denmark; Department of Infectious Medicine and Rheumatology, University Hospital of Copenhagen, COHYPCO, 2100 Copenhagen Ø, Denmark; Dalla Lana School of Public Health, University of Toronto, Toronto, Ontario Canada; Institute of Occupational Therapy, Physiotherapy and Radiography, Department of Health Sciences, Bergen University College, Bergen, Norway

**Keywords:** Joint laxity, Chronic pain, Joint pain, Rheumatic diseases, Pediatrics, Musculoskeletal system

## Abstract

**Background:**

There is some evidence that indicates generalized joint hypermobility (GJH) is a risk factor for pain persistence and recurrence in adolescence. However, how early pain develops and whether GJH without pain in childhood is a risk factor for pain development in adolescence is undetermined. The aims for this study were to investigate the association between GJH and development of joint pain and to investigate the current GJH status and physical function in Danish adolescents.

**Methods:**

This was a longitudinal cohort study nested within the Copenhagen Hypermobility Cohort. All children (n = 301) were examined for the exposure, GJH, using the Beighton test at baseline at either 8 or 10 years of age and then re-examined when they reached 14 years of age. The children were categorized into two groups based on their number of positive Beighton tests using different cut points (i.e. GJH4 defined as either < 4 or ≥ 4, GJH5 and GJH6 were similarly defined). The outcome of joint pain was defined as arthralgia as measured by the Brighton criteria from the clinical examination. Other outcome measures of self-reported physical function and objective physical function were also collected.

**Results:**

Children with GJH had three times higher risk of developing joint pain in adolescence, although this association did not reach statistical significance (GJH5: 3.00, 95% [0.94-9.60]). At age 14, the adolescents with GJH had significantly lower self-reported physical function (for ADL: GJH4 p = 0.002, GJH5 p = 0.012; for pain during sitting: GJH4 p = 0.002, GJH5 p = 0.018) and had significantly higher body mass index (BMI: GJH5 p = 0.004, GJH6 p = 0.006) than adolescents without GJH. There was no difference in measured physical function.

**Conclusion:**

This study has suggested a possible link between GJH and joint pain in the adolescent population. GJH was both a predictive and a contributing factor for future pain. Additional studies with larger sample sizes are needed to confirm our findings.

## Background

Musculoskeletal disorders are often characterized by pain and physical impairment. This may influence the quality-of-life of an individual, which could cause an economic burden to the society [1,2]. Generalized joint hypermobility (GJH) is one of the musculoskeletal disorders, and is defined by a certain number of positive joint mobility tests [3]. Further, GJH is part of the diagnostic criteria for benign joint hypermobility syndrome (BJHS) [4]. Prevalence of GJH varies according to age, sex and ethnicity. It also varies based on the diagnostic criteria used and the reliability of the joint mobility test [5]. Generally, a threshold of four or more positive joints out of 9 possible using the Beighton tests (GJH4) is used to determine GJH for adults [3]. However, to date there are no consensus criteria for GJH in children. Since joint laxity decreases with age [5], a higher number of positive Beighton tests has been suggested as a diagnostic criteria for children, (i.e. ≥6 positive Beighton tests (GJH6) for 10–12 years) [6]. The prevalence of GJH4 for children has been estimated to be between 29% to 35%, whereas the prevalence of GJH6 has been reported to be between 9% to 11% [7,8].

The relationship between musculoskeletal complaints and GJH has been investigated in a few studies, but the studies either indicated a relationship [9-11] or were unable to confirm this [12,13]. GJH has been hypothesized to be a risk factor for developing musculoskeletal pain, but it is unknown how early this pain develops. Children at 10 years with GJH and musculoskeletal pain have increased risk of pain persistence and pain recurrence in adolescence [9,10], but whether GJH without pain in childhood is a risk factor for pain development in adolescence is unclear. There is a need to increase the knowledge about when pain develops, in whom it develops, and how it may impact on physical functioning for adolescents. This information will be useful for developing preventive strategies for children with GJH [14,15].

The connection between GJH and physical functioning has been investigated. Some studies have shown an association between GJH with neuromuscular and motor development dysfunction [16-18] as explained by a poor proprioception [19,20]. Other studies have found conflicting evidence where children with GJH had a higher vertical jump height, had better static balance, had faster speed skills, and faster reaction skills than children without GJH [7,8].

The current study had two aims. The first was to investigate the association between GJH and development of joint pain in adolescents. The second was to investigate the current GJH status and self-reported physical functioning and objectively measured physical function by re-examination, respectively, six and four years after the enrolment.

## Methods

This study was a cohort study [21,22] within the Copenhagen Hypermobility Cohort (COHYPCO).

### Procedures

This study was a continuation of two cross-sectional surveys of a representative sample of preadolescent Danish school children. The surveys took place at two different municipalities in the rural area of Greater Copenhagen, Denmark: 1) the Ballerup and 2) Taarnby municipalities. The children in the Ballerup cohort were examined at eight years of age in 2006, and the children in the Taarnby cohort were examined at ten years of age in 2008. The two cohorts together formed the COHYPCO [7,8].

In 2012, the children and their parents were re-invited to participate in the COHYPCO study by an information letter sent through the online school communication system. Parents, children and their teachers were invited to an information meeting that was held in the two municipalities. The children were examined at school from November to December 2012. Children who were on sick-leave or on vacation were either examined in January 2013 or in April-May 2013.

The Regional Committees on Health Research Ethics for Southern Denmark did not consider this study to be invasive and therefore, no ethics approval was warranted. Parents of each participating child gave their informed consent according to the Declaration of Helsinki [23], and before examination each child gave oral assent to participate.

### Study population

**Figure 1:**
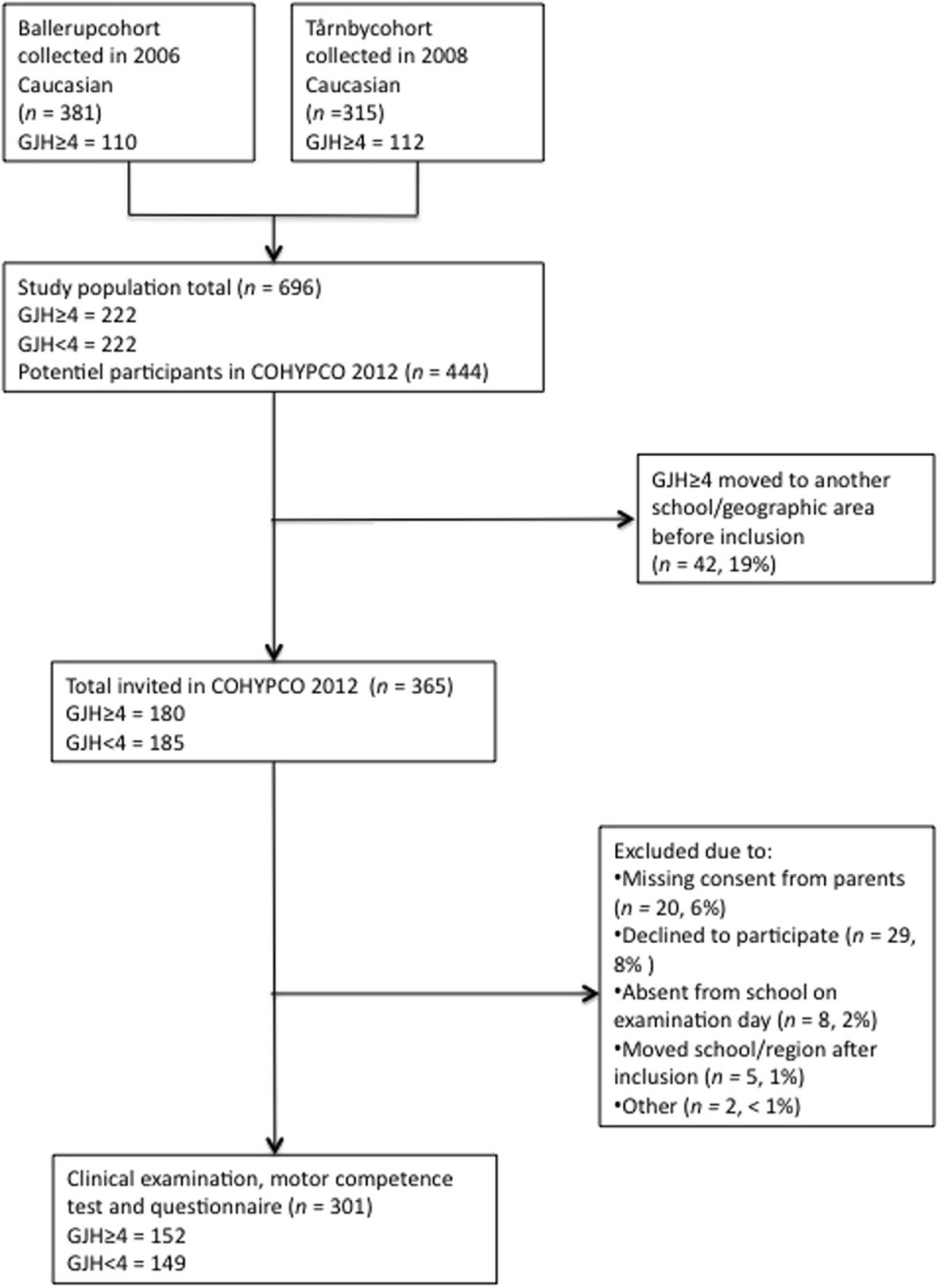
**Flowchart of children included in the study.**

Participants for this study were selected according to their GJH status and pain status at baseline. All children of Caucasian origin, with no pain at baseline, and categorized as ≥ GJH4 (n = 222) at baseline were defined as cases (Figure 1). Age- and sex-matched controls were randomly chosen on a ratio of 1:1 from Caucasian children (within the same class) who were categorized as < GJH4 (n = 222) at baseline. At follow-up, all participants were in the eighth grade, except for one who was in the seventh grade. Fifteen different public schools in the two municipalities participated.

### Measurements

#### Clinical examination

The clinical and motor competence examination took place at each school during school-time. The children were not allowed any stretching or warm-up before testing. They were tested in groups of three to four. The duration of examination varied from 45 to 60 minutes for each group and was performed by four examiners. One examiner (one of the two medical doctors (MD’s)) was responsible for the clinical examination and two of the motor competence tests (i.e. dynamic balance and muscle explosive force), one examiner (physiotherapist (PT)) was responsible for the third motor competence test (i.e. static balance), one examiner was responsible for administering the questionnaire (PT), and the last examiner was responsible for the logistics and communication between players. All examiners, who were trained thoroughly in carrying out the test battery, were mutually blinded to each other’s results and to the baseline GJH status. The same clinical examination tests and criteria used in the baseline, previously shown to have high inter-examiner reproducibility for diagnosing GJH and BJHS, kappa values of 0.74 and 0.84 [24], were used in the follow-up.

### Motor competence

The three motor competence tests focused on motor competence in the lower extremities (i.e. static balance, dynamic balance and muscle explosive force). The children were allowed to practice the actual motor competence tests for three times before being tested.

Static balance comprised of testing postural sway in three different standing balance tasks on a Wii Balance Board (WBB) (Nintendo, Kyoto, Japan) [25]. These balance tests were as follows: Romberg test with eyes open, Romberg test with eyes closed, and one-leg stance (on dominant leg) with eyes open [26]. The children stood with bare feet on the balance board, arms crossed over their chest, and were instructed to remain as still as possible for the whole trial of 30 seconds. Sampling frequency was 20 Hz. Romberg open eyes test was measured one time for familiarization and the two remaining balance tests were repeated three times. The averages for these were used to calculate the following parameters: 95% confidence ellipse area of the centre of pressure (in cm^2^), anterior-posterior displacement (in cm), medial-lateral range displacement (in cm) and centre of pressure path length (in mm). These tests have been found to have satisfactory reproducibility for a children aged 10–14 [27].

Dynamic balance was measured using the zig-zag jumping test from Movement ABC-2 [28], which recently has been found to be a valid instrument for measuring activities in children [29]. The children performed barefoot one-legged jumping on six mats positioned in a zig-zag row. The number of correct consecutive jumps from the start (maximum 5) without resting was noted. The children had one practice attempt with each leg. If the maximum number of jumps was achieved in the first attempt, there were no more additional attempts; otherwise, the test was performed a maximum of twice per leg (scoring 0–6). The maximum score of six was only achieved for 5 consecutive jumps in the first trial. The worst score (0) was recorded if no jumps were performed. The best score for each leg was selected.

Muscle explosive force was measured using the child’s height and vertical jump on two legs (i.e. Abalakov’s test). This is a widely used test to investigate explosive strength or power, but to our knowledge reliability or validity has not been documented in children or adolescents [30]. The highest jump out of three attempts was selected [8].

### Questionnaire

On the day of the examination, the Rheumatoid and Arthritis Outcome Score for children (RAOS-child version 1) questionnaire was filled out electronically by each child. This questionnaire was developed for children and it is in the same format as the Knee Osteoarthritis Outcome Score for children (KOOS-child). The KOOS-child has been validated in children aged 10–12 years, but only covers the knee [31]. The RAOS-child questionnaire consists of questions about physical functioning for three body parts: the knee, hip and ankle. Similar modifications have been done to the KOOS questionnaire for adults [32], called RAOS [33] which has been found to be a valid, reliable and responsive outcome measurement. These properties have not been tested for the RAOS-child, but it is assumed that the questionnaire has similar properties as the adult version. RAOS-child contains five domains: symptoms, pain, activities of daily living (ADL), sport and quality-of-life (QOL). There are 46 questions. Each question has 5 response categories, scored from 0 to 4 (0 = none, 1 = mild, 2 = moderate, 3 = severe, 4 = extreme). The total score for each dimension is calculated as follows [31]:

$$ 100\ \mathrm{minus}\ \mathrm{average}\ \left(\mathrm{of}\ \mathrm{t}\mathrm{hat}\ \mathrm{dimension}\right)/4\ *100,\ \mathrm{meaning}\ 100\ \mathrm{is}\ \mathrm{equal}\ \mathrm{t}\mathrm{o}\ \mathrm{normal}\ \mathrm{function} $$

Additional questions on musculoskeletal health in relation to prior injuries (‘Have you experienced dislocation or subluxation in one joint?’ yes/no; ‘Have you experienced epicondylitis, tenosynovitis or bursitis?’ yes/no), physical activity (‘Do you do any sports in your spare time?’ yes/no; ’At what level are you practising your primary sports activity?’ Elite/sub elite/exercise level; ‘How many hours a week are you practicing your primary sports activity?’). Subjective pain disabilities (SPD) were also included in the questionnaire. These questions have shown to have high reliability in a population of school children in third and fifth grade (kappa = 0.9) [6].

### Measurements for exposure, outcome and confounders

Beighton scores at baseline and follow-up were used as independent variables for the exposure GJH. Data was reported using three different definitions based on the number of positive Beighton tests. Definition 1: <GJH4 versus (vs) ≥ GJH4 (Beighton score of 4) [3], definition 2: <GJH5 vs. ≥GJH5 (Beighton score of 5) [6], and definition 3: <GJH6 vs. ≥GJH6 (Beighton score of 6) [7,8]. The Brighton criterion regarding arthralgia (i.e. pain in more than four joints for more than three months) measured at follow-up was used as dependent factor for joint pain.

For the association between GJH at baseline and joint pain at follow-up, age and sex at baseline were tested as potential confounders. For the association between current GJH status and joint pain, the following variables at follow-up were tested as potential confounders: age, sex, BMI (body mass index), previous lower limb injuries, physical activity and motor competence.

### Data analysis and statistics

Descriptive statistics were summarized using either frequency tables or means/medians. Data was reported by the three classifications with respect to the number of positive Beighton tests. Group differences in demography, self-reported (RAOS-child, SPD) and measured physical function (motor competence tests) were tested using independent t-test for the parametric data and either Mann–Whitney U-test, chi-square test or Fisher’s exact test for the non-parametric data. P-values less than 0.05 (two-tailed) were considered statistically significant.

An unadjusted logistic regression model was computed to determine whether GJH was a predictive and/or an associative factor for reporting joint pain. Potential baseline or follow-up confounders were individually added to the unadjusted model. If the β-coefficient of GJH changed by more than 10% this variable was considered a confounder and was included in the final multivariable logistic regression model [34]. Statistical significance required that the 95% Confidence Interval (CI) did not include 1. All analyses were performed in SPSS version 21 (IBM SPSS Inc, Chicago, IL, USA).

## Results

### Participants

In total, 301 (82% of invitees) children of Caucasian origin (median age 14.00 [range = 13–15]) completed the follow-up examination. Reasons for non-participation included: missing consent from parents, declining participation, absence from school on examination day, having moved school/region after inclusion and other reasons (such as other chronic diseases) (Figure 1). The demography for the three definitions of GJH is presented in Table 1. There was significantly higher proportion of girls than boys with GJH4 (p = 0.035) and GJH6 (p = 0.034), and GJH5 and GJH6 had statistically higher BMI than their respective control groups (GJH5: p = 0.004, GJH6: p = 0.006).

**Table 1:** **Demography by the three definitions of generalized joint hypermobility (GJH)**

	**GJH4**			**GJH5**			**GJH6**		
**Variable**	**< GJH4**	**≥ GJH4**	**p-value**	**< GJH5**	**≥ GJH5**	**p-value**	**< GJH6**	**≥ GJH6**	**p-value**
	**(n = 171)**	**(n = 130)**		**(n = 217)**	**(n = 84)**		**(n = 237)**	**(n = 64)**	
Age, median (range)	14 (13–15)	14 (13–15)	0.13	14 (13–15)	14 (13–15)	0.24	14 (13–15)	14 (13–15)	0.61
^1^BMI, mean (sd)	20.02 (2.62)	20.57 (2.77)	0.08	19.95 (2.52)	21.03 (2.98)	0.004*	20.03 (2.62)	21.07 (2.85)	0.006*
Gender, no. of girls, n (%)	75 (43.9)	73 (56.2)	**0.04** ^**a,**^ *	100 (46.1)	48 (57.1)	0.09^a^	109 (46.0)	39 (60.9)	**0.03** ^**a,**^ *
**Musculoskeletal health**, n (%)									
Arthralgia in 1–3 joints (> 3 months), (n = 301)	9 (5.3)	10 (7.7)	0.39^a^	12 (5.5)	7 (8.3)	0.37^a^	14 (5.9)	5 (7.8)	0.58^a^
Arthralgia in >4 joints (> 3 months), (n = 300)	4 (2.3)	8 (6.2)	0.14^b^	6 (2.8)	6 (7.1)	0.08^a^	7 (3.0)	5 (7.8)	0.08^a^
^2^Dislocation/subluxation, (n = 293)	10 (5.8)	9 (6.9)	0.70^a^	11 (5.1)	8 (9.5)	0.15^a^	13 (5.5)	6 (9.4)	0.26^a^
^3^Soft tissue rheumatism, (n = 293)	5 (2.9)	5 (3.8)	0.66^a^	6 (2.8)	4 (4.8)	0.47^b^	8 (3.4)	2 (3.1)	1.00^b^

^1^BMI = Body Mass Index (calculated as = bodyweight in kg/ height in m*height in m) ^2^Dislocation/subluxation is based on the question: ‘Have you experienced dislocation or subluxation in one joint’. ^3^Soft tissue rheumatism is based on the question: ‘Have you experienced epicondylitis, tenosynovitis or bursitis?’

Methods/Hypothesis testing: Age: Mann Whitney u-test; BMI (body mass index): independent t-test; Gender, musculoskeletal health: *X* ^*2*^ *,* ^a^Pearson’s chi-square; ^b^Fishers exact test. Significant difference between groups are marked with *and written with bold.

### GJH as a risk of developing or having pain

In the longitudinal analysis, children with GJH based on the GJH5 definition at baseline had a threefold increased risk for developing joint pain at follow-up, although this association did not reach statistical significance (GJH5; 3.00 [0.94-9.60]) (Table 2). There were no identified confounders for the associations for GJH5 and GJH6 and therefore, it was not possible to conduct an adjusted model.

**Table 2:** **Longitudinal data: Odds ratio (OR) for generalized joint hypermobility (GJH), being a predictive factor for pain (arthralgia) development**

	**Outcome** ^**a**^		**Univariate** ^**b**^ **analysis**	**Multivariable analysis**
	**Arthralgia (n = 12)**	**Non-arthralgia (n = 288)**	**OR (95% CI)**	**OR (95% CI)**
**Exposure**				
<GJH4^1^	5	145	1.00	1.00
≥GJH4^1^	7	143	1.42 (0.44–4.58)	1.37 (0.42–4.43)^c^
<GJH5^2^	6	216	1.00	
≥GJH5^2^	6	72	3.00 (0.94–9.60)	NC^d^
<GJH6^3^	9	241	1.00	
≥GJH6^3^	3	47	1.71 (0.45–6.55)	NC^d^

^1^< GJH4 versus ≥ GJH4 = 3 versus 4 or more positive Beighton tests out of a maximum of 9 Beighton tests ^2^< GJH5 versus ≥ GJH5 = 4 versus 5 or more positive Beighton tests out of a maximum of 9 Beighton tests ^3^< GJH6 versus ≥ GJH6 = 5 versus 6 or more positive Beighton tests out of a maximum of 9 Beighton tests.

^a^Outcome (arthralgia) measured at follow-up at 14 years old, exposure (GJH) measured at baseline at eight or ten years old (cohort study). ^b^Univariate model. ^c^Multivariable model adjusted to gender. ^d^No confounders identified for this association and no multivariable models conducted. NC = not conducted.

In the unadjusted logistic regression analysis, children with GJH (independent of cut-off level) had three times higher risk of reporting joint pain at follow-up, although this association did not reach statistical significance (OR [95% CI]; GJH4: 2.76 [0.81-9.38], GJH5: 2.96 [0.84-8.60], GJH6: 2.77 [0.85-9.05]) (Table 3). Controlling for potential confounders did not change these results.

**Table 3:** **Odds ratio (OR) for generalized joint hypermobility (GJH) being a contributing factor for pain (arthralgia) reporting**

	**Outcome** ^**a**^		**Univariate** ^**b**^ **analysis**	**Multivariable analysis**
	**Arthralgia (n = 12)**	**Non-arthralgia (n = 288)**	**OR (95% CI)**	**OR (95% CI)**
**Exposure**				
<GJH4^1^	4	167	1.00	1.00
≥GJH4^1^	8	121	2.76 (0.81–9.38)	2.16 (0.61–7.64)^c^
<GJH5^2^	6	210	1.00	1.00
≥GJH5^2^	6	78	2.69 (0.84–8.60)	2.38 (0.66–8.60)^d^
<GJH6^3^	7	229	1.00	1.00
≥GJH6^3^	5	59	2.77 (0.85–9.05)	2.36 (0.61–9.10)^e^

^1^< GJH4 versus ≥ GJH4 = 3 versus 4 or more positive Beighton tests out of a maximum of 9 Beighton tests ^2^< GJH5 versus ≥ GJH5 = 4 versus 5 or more positive Beighton tests out of a maximum of 9 Beighton tests ^3^< GJH6 versus ≥ GJH6 = 5 versus 6 or more positive Beighton tests out of a maximum of 9 Beighton tests.

^a^Outcome (arthralgia) and exposure (GJH) measured at follow-up at 14 years old (cross-sectional). ^b^Univariate model. ^c^Multivariable model adjusted to gender, sway. ^d^Multivariable model adjusted to gender, previous lower limb injuries (yes/no), sway. ^e^Multivariable model adjusted to gender, previous lower limb injuries (yes/no), vertical jump, sway.

### Self-reported and measured physical function at follow-up

Self-reported ADL as reported in the RAOS-child questionnaire was significantly lower (poorer) in the children with GJH (i.e. GJH4 (p = 0.002) and GJH5 (p = 0.012)) (Table 4). For the SPD, there was significantly higher proportion of GJH children reporting disturbing pain while sitting in class for GJH4 (p = 0.002) and GJH5 (p = 0.018).

**Table 4:** **Self-reported physical function and physical activity for the three definitions of generalized joint hypermobility (GJH) for children at the age of 14**

	**GJH4**			**GJH5**			**GJH6**		
**Variable**	**< GJH4**	**≥ GJH4**	**p-value**	**< GJH5**	**≥ GJH5**	**p-value**	**< GJH6**	**≥ GJH6**	**p-value**
	**(n = 171)**	**(n = 130)**		**(n = 217)**	**(n = 84)**		**(n = 237)**	**(n = 64)**	
^**1**^ **RAOS-child** , mean (sd)									
Symptoms (n = 299)	88.55 (11.04)	86.10 (12.51)	0.16	87.82 (11.80)	86.67 (11.60)	0.39	87.73 (11.90)	86.68 (11.15)	0.34
Pain (n = 293)	89.53 (10.08)	87.01 (10.81)	0.02*	88.97 (10.31)	87.09 (10.80)	0.10	88.77 (10.56)	87.24 (10.07)	0.12
ADL (n = 293)	96.00 (6.22)	94.47 (5.84)	0.002*	95.70 (6.17)	94.44 (5.84)	0.012*	95.54 (6.20)	94.64 (5.67)	0.06
Sport (n = 296)	87.59 (14.08)	84.31 (16.57)	0.07	86.62 (14.91)	85.09 (16.14)	0.42	86.37 (15.17)	85.60 (15.61)	0.56
QOL (n = 297)	82.57 (14.45)	78.52 (17.47)	0.06	81.36 (15.66)	79.42 (16.61)	0.30	81.00 (16.10)	80.16 (15.37)	0.43
**Subjective Pain Disabilities** (SPD), n (%)									
Pain disturbing sleeping (n = 299)	10 (5.8)	9 (7.0)	0.68	15 (6.9)	4 (4.9)	0.52	15 (6.4)	4 (6.3)	1.00
Pain disturbing sitting during class (n = 298)	11 (6.4)	23 (18.1)	0.002*	19 (8.8)	15 (18.5)	0.018*	24 (10.2)	10 (16.1)	0.19
Paint disturbing walking > 1 km (n = 297)	26 (15.2)	25 (19.8)	0.30	36 (16.6)	15 (18.8)	0.66	40 (16.9)	11 (18.0)	0.84
Pain disturbing physical exercise class (n = 299)	35 (20.5)	36 (28.1)	0.12	49 (22.6)	22 (26.8)	0.44	56 (23.7)	15 (23.8)	0.99
Pain disturbs hobbies (n = 299)	27 (15.8)	28 (21.9)	0.18	38 (17.5)	17 (20.7)	0.52	42 (17.8)	13 (20.6)	0.61
**Physical activity**									
^2^Sports active, *leisure time*, (n = 298), n (%)	137 (80.6)	105 (82.0)	0.75	173 (80.1)	69 (84.1)	0.42	190 (80.9)	52 (82.5)	0.76
^3^Activity level (n = 245), n (%)			0.88			0.25			0.13
*Elite*	32 (22.9)	22 (21.0)		40 (22.7)	14 (20.3)		44 (22.8)	10 (19.2)	
*Sub elite*	44 (31.4)	36 (34.3)		52 (29.5)	28 (40.6)		57 (29.5)	23 (44.2)	
*Exercise level*	64 (45.7)	47 (44.8)		84 (47.7)	27 (39.1)		92 (47.7)	19 (36.5)	
^4^Hours per week (n = 299), median (range)	3.00 (0–23)	3.75 (0–27)	0.70	3.00 (0–23)	4.00 (0–27)	0.25	3.00 (0–23)	4.00 (0–27)	0.12

^1^RAOS score, with 100 indicating no problems and 0 indicating severe problems. ^2^Sports active is based on the question: ‘Do you do any sports in you spare time?’ Rated as yes/no. ^3^Activity level is based on the question: ‘At what level are you practising you primary sports activity?’ With the answering categories: Sub elite, elite or exercise level. ^4^Hours per week is based on the question: ‘How many hours a week are you practicing your primary sports activity?’ Measured as the group average.

Methods/Hypothesis testing: RAOS, Physical activity (hours per week): Mann–Whitney u-test; SPD, Physical activity: *X* ^*2*^ (Pearson’s). Significant difference between groups are marked with *and written with bold.

Children with GJH did not perform better in motor competence, neither in static (sway) nor dynamic balance (zig-zag jump), than children without GJH (Table 5). Children with GJH had a lower vertical jump height; however, the difference was not statistically significant (GJH4 p = 0.33, GJH5 p = 0.15, GJH6 p = 0.12).

**Table 5:** **Measured motor competence for the three definitions of generalized joint hypermobility (GJH) at follow-up**

	**GJH4**			**GJH5**			**GJH6**		
**Motor competence**	**< GJH4**	**≥ GJH4**	**p-value**	**< GJH5**	**≥ GJH5**	**p-value**	**< GJH6**	**≥ GJH6**	**p-value**
	**(n = 171)**	**(n = 130)**		**(n = 217)**	**(n = 84)**		**(n = 237)**	**(n = 64)**	
^**1**^ **Zigzag hop**, *no. of consecutive hops,* median (range)									
Right leg (n = 300)	6 (0–6)	6 (2–6)	0.72	6 (0–6)	6 (3–6)	0.07	6 (0–6)	6 (3–6)	0.018*
Left leg (n = 298)	6 (0–6)	6 (2–6)	0.67	6 (0–6)	^11^6 (2–6)	0.77	6 (0–6)	6 (2–6)	0.74
**Vertical jump,** *cm*, (n = 299), mean (sd)	32.31 (6.42)	31.46 (6.86)	0.33	32.31 (6.33)	31.00 (7.26)	0.15	32.30 (6.29)	30.62 (7.59)	0.12
**Sway,** mean (sd)									
**Romberg open eyes** (n = 300)									
^2^95% areal, *cm* ^*2*^	4.57 (2.26)	5.11 (2.42)	0.05	4.72 (2.24)	5.01 (2.61)	0.72	4.73 (2.26)	5.10 (2.62)	0.45
^3^Anterior-posterior range, *cm*	2.53 (0.73)	2.67 (0.84)	0.24	2.56 (0.75)	2.67 (0.85)	0.39	2.57 (0.75)	2.68 (0.90)	0.51
^4^Medial-lateral range, *cm*	2.56 (0.67)	2.66 (0.75)	0.48	2.61 (0.70)	2.57 (0.72)	0.34	2.61 (0.70)	2.58 (0.73)	0.54
^5^Centre of pressure path length, *mm*	56.13 (11.74)	56.53 (9.23)	0.46	56.10 (11.15)	56.83 (9.53)	0.39	56.24 (10.99)	56.54 (9.68)	0.74
**Romberg closed eyes** (n = 300)									
^2^95% areal, *cm* ^*2*^	8.83 (3.94)	9.39 (5.43)	0.56	8.85 (3.78)	9.63 (6.35)	0.82	9.08 (4.74)	9.02 (4.26)	0.87
^3^Anterior-posterior range, *cm*	3.62 (0.86)	3.67 (1.13)	0.88	3.61 (0.85)	3.72 (1.26)	0.94	3.66 (1.03)	3.57 (0.83)	0.53
^4^Medial-lateral range, *cm*	3.78 (0.92)	3.92 (0.90)	0.20	3.82 (0.90)	3.90 (0.95)	0.61	3.85 (0.95)	3.82 (0.77)	0.85
^5^Centre of pressure path length, *mm*	85.55 (24.25)	85.17 (18.69)	0.59	84.95 (22.73)	86.52 (20.04)	0.30	85.64 (23.34)	84.40 (16.07)	0.79
**One leg stance** (n = 298)									
^2^95% areal, *cm* ^*2*^	9.80 (3.19)	10.13 (3.73)	0.80	9.90 (3.19)	10.04 (4.02)	0.61	10.01 (3.37)	9.67 (3.69)	0.18
^3^Anterior-posterior range, *cm*	4.54 (1.09)	4.63 (1.19)	0.73	4.58 (1.13)	4.57 (1.15)	0.88	4.61 (1.15)	4.44 (1.05)	0.17
^4^Medial-lateral range, *cm*	3.25 (0.56)	3.27 (0.61)	0.57	3.27 (0.55)	3.24 (0.65)	0.18	3.27 (0.57)	3.23 (0.63)	0.11
^5^Centre of pressure path length, *mm*	132.26 (37.19)	129.23 (32.72)	0.54	132.72 (37.28)	126.27 (29.19)	0.33	132.68 (37.02)	124.34 (27.11)	0.15

^1^Zigzag hop measured on a scale from 0–6, where 6: 5 consecutive jumps in first trial; 5: 5 consecutive jumps in second trial; 4: maximum of 4 consecutive jumps; 3: maximum of 3 consecutive jumps; 2: maximum of 2 consecutive jumps; 1: maximum of 1 consecutive jumps; 0: maximum of 0 consecutive jumps. ^2^95% confidence ellipse area of the Centre of Pressure (cm^2^) ^3^Anterior-posterior displacement (cm) ^4^Medial-lateral range displacement (cm) ^5^Centre of pressure path length (mm).

Methods/Hypothesis testing: Mann–Whitney U-test. Significant difference between groups are marked with *and written with bold.

## Discussion

The result of this study suggested that GJH5 without pain in childhood at eight or ten years of age is a possible predictive factor for developing joint pain in adolescence, although this association did not reach the predefined level of statistical significance. It also indicated that there was a positive association between GJH and experiencing joint pain at 14 years of age. Furthermore, we found that adolescents aged 14 years with GJH5 or GJH6 had significantly higher BMI and self-reported lower physical functioning. They also experienced daily pain more frequently.

The association between GJH in childhood and development of joint pain in adolescence is partly in accordance with findings from previous studies. Other studies have found that hypermobility was a significant predictor for pain recurrence and for pain persistence at the age of 14 and/or 16 at follow-up [10,35], but was not a predictor for pain incidence one year later [36]. More clearly, the current study proposes that GJH is a predictor (close to reaching significance) for incident joint pain at six and four years follow-up, indicating an increased risk for GJH with no pain at baseline. There have been no other studies that has reported this. Although a recent study found an increased risk of pain at 18 years of age in children who had GJH at 14 years of age. Unfortunately, pain status in GJH at baseline was not reported [11].

We also found that GJH seemed to be a contributing factor for having joint pain at 14 years of age. This association was not apparent in the baseline cross-sectional studies of our population when they were aged either eight or ten, where no relation between GJH and musculoskeletal pain was found. This has been reported in other studies of children in that age range [6-8]. This means that an association at early age is possibly not present. Our current results suggest that the impact of GJH starts later, somewhere between ages 10 and 14, or at least at 14 years of age with such relationship approaching significance in the current longitudinal analysis.

At baseline, there was an equal distribution of children being < GJH4 vs. ≥GJH4 and between boys and girls. The number of children with ≥ GJH4 from baseline to follow-up had decreased, supporting that joint laxity is decreasing by increasing age [5].

In the current cross-sectional study, adolescents with GJH also reported a lower self-reported physical function. In a previous study, the self-reported SPD was not associated with GJH [6], but a higher SPD score was associated with musculoskeletal pain or pain persistence/recurrence in children [9,37]. Since GJH in the current study was associated with pain, it could be likely that children with GJH reported lower self-reported physical functioning due to pain.

At baseline, children with GJH had better motor competence than their classmates (i.e. jump, precision tasks) [7,8]. However, at follow-up, GJH did not perform better which may indicate that GJH during the follow-up period may have influenced motor competence negatively or that the tests were not precise/challenging enough to differ between the groups.

The estimates for GJH as a contributing factor for having joint pain were the same for the three different definitions. This may indicate that the different cut-off levels for the number of positive joints had no influence on the data.

The estimates for both the cross-sectional as well as for the longitudinal analyses have wide confidence intervals. This affects the statistical power of the results negatively and weakens the association between GJH and developing joint pain. The small sample size and low number of outcome events must be an explanation for this and why these associations should be confirmed in a larger study. Further to this, the small sample size may explain the inconsistent pattern of the longitudinal analysis where GJH5 had the highest OR followed by GJH6 and GJH4.

Including age groups of both eight and ten years at baseline could be a limiting factor due to the shorter follow-up period for one of the groups. Therefore, this could have weakened the association of having GJH as a child and developing joint pain in adolescence. However, this was not confirmed, since there was an increased risk in children at ten compared with eight years at baseline. Due to the relatively small groups they were pooled into one large group at follow-up. Taken together, despite the small number of baseline measurements and outcome events, we saw an increased risk of pain development in GJH, suggesting an association between GJH and joint pain for adolescents who had no pain at baseline.

Selection of a limited number of control subjects was based on a desire to achieve an equal number of exposure contrasts, knowing that it could have caused systematic selection bias. However, since selection criteria were based on exposure (GJH) and not outcome status (joint pain), this is unlikely to have biased this association [21].

Measurement of outcome status (conducted by medical history) from the clinical examination was more likely to be confounded by recall bias than the exposure status. But since the current outcome (pain in more than 4 joints for more than 3 months) is a relatively “hard” outcome, it is not likely to have biased this association.

Another weakness of this study was the lack of a full baseline dataset on potential confounders. Although we did investigate and adjust for potential confounders, there may have been residual confounding not accounted for because we had no information about the following: injuries at baseline, family history of rheumatic diseases or socioeconomic status.

The strengths of this study were having clinical examinations performed at both baseline and follow-up. This strengthens the validity of the exposure and outcome, since the exposure is measured objectively and is therefore free of recall bias, and the outcome is a relatively hard end-point. The examiners performing the clinical tests were the same as in the baseline studies. Each examiner tested a random number of children at baseline and at follow-up, meaning that they did not test the same child at both test rounds. It is therefore assumed that the examiners were blinded to the health status of the child. The examiner blindness also minimizes non-differential misclassification of both the exposure and outcome status.

## Conclusion

This study suggests a possible link between GJH in childhood and joint pain in adolescence. Children at eight or ten years of age with GJH5 and no pain at baseline were found to have a threefold increased risk of developing pain at 14 years of age. Although this association did not reach the predefined level of statistical significance future studies with a bigger sample size are needed to confirm these findings.

Furthermore, adolescents at 14 years of age with GJH have higher BMI, lower self-reported physical function and experience daily pain more frequently, but GJH does not seem to influence measured physical function at 14 years of age.

## Abbreviations

BJHS: Benign joint hypermobility syndrome
COHYPCO: Copenhagen hypermobility cohort
EDS: Ehlers-Danlos syndrome
GJH: Generalized joint hypermobility
RAOS-child: Rheumatoid and arthritis outcome score for children
SPD: Subjective pain disabilities
